# Oral Motor Treatment Efficacy: Feeding and Swallowing Skills in Children with Cerebral Palsy

**DOI:** 10.1155/2021/6299462

**Published:** 2021-10-25

**Authors:** Maria E. Widman-Valencia, Luis F. Gongora-Meza, Héctor Rubio-Zapata, Rita E. Zapata-Vázquez, Elma Vega Lizama, Marco Ramírez Salomón, Damaris Estrella-Castillo

**Affiliations:** ^1^Academic Body of Public Health, School of Medicine, Autonomous University of Yucatan, 97000, Mexico; ^2^Academic Body of Endodontic, Dental School, Autonomous University of Yucatan, 97000, Mexico

## Abstract

This study is aimed at identifying the relationship between oral motor treatment and the improvement of abilities for feeding and swallowing in boys and girls with CP residing in the state of Yucatán. The sample consisted of 30 patients with a diagnosis of CP and the presence of ADT, with gross motor function levels from II to V, between 3 and 14 years old, of which 50% received oral motor treatment. The predominant diagnosis was spastic CP and tetraplegia. An interview was carried out with the tutor, the application of the gross motor skills scale, and an assessment of feeding skills. The feeding and swallowing skills that improved significantly with the oral motor treatment were mandibular mobility, tongue activity, abnormal reflexes, control of breathing, and general oral motor skills (*p* ≤ 0.05). Within the sample that did not receive oral motor treatment, 46% presented low or very low weight and 40% referred recurrent respiratory diseases. In the end, it was concluded that feeding skills improve significantly with oral motor treatment, regardless of the severity of gross motor involvement. Likewise, oral motor treatment was associated with a lower presence of respiratory diseases and nutritional compromise.

## 1. Introduction

Cerebral palsy (CP) is the most common cause of severe physical disability and motor function deterioration in children. Its prevalence has been increasing as more children survive the neonatal stage and the worldwide incidence is estimated at 2 per 1000 births [1–3]. Motor disorders originated by CP are often accompanied by sensory, perception, cognition, communication, and behavior disorders, which are usually aggravated by other diseases such as epilepsy or malnutrition. Feeding and swallowing disorders (FSD) are the most common in children with CP. Swallowing is a complex sensory-motor process that coordinates the bilateral contraction/relaxation in the muscles of the mouth, tongue, larynx, pharynx, and esophagus, traditionally subdivided into four phases: preoral, oral, pharyngeal, and esophageal [4]. Dysfunction of the pharyngeal phase is very dangerous due to the increased risk or incidence of food aspiration in the airways leading to recurrent lung infections and, in severe cases, death by bronchoaspiration. The clinical effect of preoral and oral phases disorders are often chronic, such as malnutrition or recurrent infections, which is very common in children with CP [5–7].

Oral motor rehabilitation therapy in children with CP aims at reducing or eliminating swallowing disorders and promoting functional feeding [8]. Some studies suggest that intervention with oral motor therapy (OMT) has a beneficial effect on functional independence levels and improves the quality of life [9, 10] of patients with FSD; for example, after 8 weeks of OMT, it has been proved that the body mass index increases while the use of feeding tubes reduces [11–13]. This proves the importance of OMT in patients with FSD. In this study, the purpose was to compare the feeding and swallowing skills in Yucatecan children that had received OMT with those who had not.

## 2. Materials and Methods

### 2.1. Subjects

Thirty children with CP diagnosis were included, with ages between three and fourteen years old (8 ± 4.07), fourteen males (47%) and sixteen females (53%), which had been registered in physical rehabilitation centers in the city of Merida, Yucatan. All participants presented some FSD, according to the Eating and Drinking Ability Classification System (EDACS) [14], and attended physiotherapy sessions at the different centers they belonged to for at least one year before this study was conducted; some of them participated in OMT in the past and some other did not. All participants received oral feeding only.

### 2.2. Study Design

The research protocol was approved by the Ethics Committee of the Faculty of Medicine attached to the Universidad Autónoma de Yucatán. Necessary authorizations were obtained at the following associations: Solyluna A.C., Construyendo Sonrisas: Patronato Peninsular Pro Niños con Deficiencia Mental A.C., Fundación de Orientación Holística, and Centro de Rehabilitación e Inclusión Infantil Teletón, CRIT Yucatán. The subjects were collected through nonprobability sampling by quota. The study population was divided into two groups: (1) those who had received OMT uninterruptedly in the association that they attended to for at least one year before the evaluation, on any of the physical therapy approaches (manual therapy, electrotherapy, active-assisted exercises, etc.), and (2) those who had not received any kind of OMT.

For this study, children receiving OMT attended two types of sessions in concordance with their individual therapeutic goals: passive range of motion exercises and active-assistive range of motion exercises. They received also sensory stimulation of the muscles of the face and mouth, with different textures and therapeutic devices. OMT sessions took place 2–3 times per week, with a maximum duration of 30 minutes, and were conducted by both occupational and physical therapists specializing in language and speech therapy.

A minimum attendance prerequisite was set at 80% of all OMT sessions to be classified as part of the first group. This requirement was verified by double checking the files of the participants provided by the different associations cited above. On the other side, all participants who were not able to prove the 80% minimum attendance requirement at their OMT sessions, along with those who stopped going to their physical rehabilitation center, those who received OMT for less than a year, and those whose rehabilitation center did not provide OMT, were classified at the group who did not receive OMT.

Parents or tutors who agreed that the minors participate in this study signed the informed consent and were interviewed during the days the children attended therapy in their rehabilitation centers. Assessments were conducted on the same day, with the parents present; the children's gross motor control was evaluated with the Gross Motor Function Classification System or GMFCS [15] and the feeding and swallowing skills (FSS) with the Feeding Oral Motor Scale [16]. GMFCS was used as a reference to compare the groups with high and low motor function. The Feeding Oral Motor Scale assesses qualitatively seven feeding skills and a global score (general oral motor skills), each one of the seven skills has one or more dimensions that get graded from 1 to 3 (optimal, average, and poor), and a higher score means more limitation.  Table 1 illustrates the seven skills and their respective dimensions.

The body mass index (BMI) (kg/m^2^) was used as an anthropometric guide of the children's nutritional status. To achieve this, weight measures were made with a SECA model 750 brand portable floor scale with a capacity of 150 kg and the height was determined with a SECA brand model 213 stadiometer. Anthropometric measurements were taken by the nutritionist at the physical rehabilitation center where the children attended. Once the anthropometric measurements were obtained, the BMI was calculated and classified, according to the BMI-for-age percentile charts adapted to children with CP accordingly to their gross motor level (GMFCS), elaborated by the Developmental Medicine and Child Neurology Journal [17], to determine if they classified as adequate weight, underweight, or very underweight within the characteristics of the CP population. The nutritional status was analyzed using the following cut points: very low weight with 12.8 or less, underweight from 12.9 to 14, adequate weight from 14.1 to 23, and overweight greater than 23.1.

Frequent respiratory disease variable was obtained by interviewing the tutor, and it was considered positive if, in a period of 6 months prior to the date of the study, the child had at least two of the following diseases: flu and common cold, bronchitis, pneumonia, airway infections, and acute symptoms of chronic diseases such as asthma, rhinitis, or rhinosinusitis.

### 2.3. Statistics Analysis

Result analyses were performed with the statistical package IBM SPSS, version 2.0. Student's *t*-tests were performed to compare the scores obtained on the different scales used to assess feeding and swallowing skills (FSS). Two-proportion *Z* tests were also performed to associate the percentages obtained within the optimal, average, and poor categories in the group that received OMT and in the one that did not. The Fisher test was used to associate nutritional status and the presence of respiratory diseases with OMT.

## 3. Results and Discussion

### 3.1. Results

This study included data from 30 children with CP (53% females and 47% males), with an average age of 8 ± 4.07 years. The group that had previously received OMT was conformed of 8 men and 7 women, with ages between 3 and 14 years (8 ± 4). On the other hand, children without treatment were 6 men and 9 women, aged 3 to 14 years (7 ± 4.15). No statistical differences were found between both groups (*p* = 0.77 and *p* = 0.75, respectively).

Initially, the two study groups, with and without OMT, are portrayed, according to the CP clinical classification and the degree of gross motor impairment. In accordance with the CP clinical classification, 67% of the population had spastic CP and 33% other classifications (ataxic 6.7%, hypotonic 13.3%, and mixed 13.3%), and according to the topographic classification, 73% had tetraplegia, 16% diplegia, and 10% hemiplegia. On the other hand, regarding the degree of gross motor impairment and according to the GMFCS scale, 63.4% of the participants were classified as levels IV and V, which correspond to the most severe level of functional limitation. The remaining 36.6% of the population was distributed between levels II and III, which refer to a mild impairment level of gross motor function. Finally, no participant was reported in level I.  Table 2 describes these characteristics in the two study groups.

Subsequently, the FSS of both groups was evaluated. Of the seven FSS, differences were found in the following skills: jaw mobility, tongue activity, abnormal reflexes, and breathing control. Special attention is given to jaw mobility and tongue activity because their dimensions are closely related to the physical intervention of the OMT. The global score and the score by sections are shown in Table 3.

Regarding jaw mobility, the opening-closing skill was the one that was significant (*p* = 0.039). Qualitatively, 67% of the children who received OMT obtained an optimal jaw mobility score; in contrast, only 27% of the children without treatment obtained the same score (difference in proportions, *p* = 0.028).

Concerning the tongue activity, lateral tongue mobility was the only significant dimension (*p* = 0.013) of the four dimensions that belong to this skill. Qualitatively, 80% of the children who received OMT obtained an optimal tongue activity score and 40% of the children without treatment obtained the same score (difference in proportions, *p* = 0.025).

Next in order, the gross motor level averages were compared, classified according to the GMFCS as levels of high functionality (II and III) and levels of low functionality (IV and V). As can be seen in Table 4, significant differences were found between children with and without OMT at both levels of functionality.

**Figure 1:**
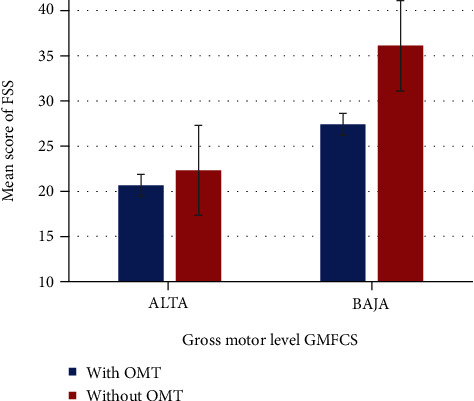
Mean score of FSS with respect to receiving OMT or not in two groups of gross motor level (high or low). A high score in FSS indicates more limitation at the skill, therefore, less desirable.

Figure 1 shows the comparison between the two levels of functionality according to the GMFCS and the average score in FSS. As can be seen, in both categories, the group that did not receive OMT obtained the highest scores, indicating a greater compromise of skills.

Finally, as a complement to the assessment of FSS, an analysis of the nutritional status of the children and the frequency with which they contract respiratory diseases was carried out. These are key signs in determining the impact of the FSD.

For the analysis of the nutritional status, the comparison of the BMI between the groups with and without OMT was made; according to the cut of the percentile curves, a BMI greater than 14 kg/m2 indicates a normal weight and a lower BMI would correspond to low or very low weight. A significant difference (*p* = 0.004) was observed in the BMI of the children who did receive treatment (15.8 ± 2.21) compared to the BMI of those who did not receive it (13.22 ± 2.37). Likewise, 93% of the children who received OMT were classified to have normal weight and only 7% were underweight. In contrast, only 54% of the children who did not receive OMT had a normal weight, while 13% and 33% manifested as being underweight and very underweight, respectively. Receiving treatment corresponded significantly to normal weight (*p* = 0.035).

Regarding the frequency with which children with CP contract respiratory diseases, a significant association was found with those not receiving OMT (*p* = 0.035).

## 4. Discussion

The main finding of this study was to demonstrate that regardless of the characteristics of the OMT, the children who had received it, at least during the last year, showed better levels of FSS compared to those children who did not receive it. The children who received OMT showed better scores in 4 of the 7 FSS: jaw mobility, tongue activity, abnormal reflexes, and breathing control. It is important to highlight that better scores in general oral motor skills were also reported. These skills were evaluated by a rehabilitation therapist in a blinded way, who did not know that the child had received or not the OMT, which improves the accuracy of the results.

Improvement in jaw stabilization found on trial groups with mouth control training indicates the beneficial effect of OMT [9]. This study showed that jaw mobility, including coordination, lateralization, and opening-closing of the jaw, obtained better scores in children with OMT. Even though abnormal oral reflexes have been observed in children with CP [18], such as phasic biting and gagging, little or no research exists that analyzes those reflexes with the OMT. However, in this study, it was found that the group with treatment had a lower presence of abnormal reflexes, compared to the group with no treatment.

Authors, such as Harden and Rydell [19], had already reported an improvement in tongue activity in children who received OMT, since they performed better according to the scale of severity of tongue protrusion, after 5 years of treatment. Likewise, by promoting proper tongue management, other problems such as tooth misalignment, excess salivation, and atypical swallowing are prevented, while other skills such as language and socialization are benefited [20].

On the other hand, other authors [21] have been interested in the effect of OMT to control breathing during feeding, improving control and coordination of swallowing and breathing, avoiding episodes of coughing or choking. However, due to the lack of a timely treatment to address the incoordination between suction and breathing, a nasogastric tube is frequently chosen, and if the weight gain is inadequate, a gastrostomy might proceed; in this sense, the importance of respiration is directly and indirectly reflected in the nutritional status; however, this point will be discussed later.

Another important finding of this study was the significant differences between children with and without OMT in the two groups of GMFCS, showing that regardless of the level of motor functionality, the OMT improves the FSS and could have even more impact in the low-function group. Some authors have shown that children with gross motor disorders tend to have a higher prevalence of FSD [22]. Other studies analyzed the relationship between the GMFCS level and the complications associated with FSD and found that for most children at level V, it is not safe to eat either solid or liquid textures, so they use semisolid textures [23, 24]. Furthermore, the benefit of OMT in children with CP may also be reflected in other areas, for example, in visual-motor coordination and language [25]; however, these functions were not explored in this study.

As previously discussed, a secondary objective of this study was to evaluate the relationship between OMT and the nutritional status of children, as well as the frequency with which they contracted respiratory diseases. Regarding the first point, it was found that the children who received OMT had higher levels of normal weight since a child who eats adequately can have a body status appropriate for his age and sex, thereby improving the chances of better function and growth. On the contrary, low weight and malnutrition favor states of immunosuppression and a greater predisposition to other diseases, which can complicate the prognosis and decrease the quality of life of children with CP. This is consistent with different studies on nutrition in children with CP, which state that eating disorders represent an important predictor of poor health, expressed in nutritional deficiencies and poor quality of life [21], which is related to the next point: the frequency of respiratory diseases. The parents of the children who had received OMT in the last year stated that their children had presented fewer events of respiratory infections, which may correspond to better FSS.

This study does not take into consideration a follow-up at home with the parents of children with OMT. Nevertheless, additional instructions regarding postures during feeding, nutrition, and adaptations were provided to the parents and they actively participated in the OMT sessions with their kids.

Also, it is important to remark that the heterogeneity of techniques of treatment, along with the different patients' diagnoses and prognoses, prevents the researchers from including only participants who received the same type of OMT. For example, some children received neuromuscular electrostimulation once a month.

Additionally, respiratory physiotherapy was not defined as a variable of the OMT for the purposes of this study.

Also, since the results were based on a limited number of subjects and due to the lack of studies in the field of OMT efficacy in children with CP, the results of this study must be treated with caution.

Nevertheless, the above-cited findings are substantial and should encourage further studies on a larger scale. One possibility could be exploring the clinical effects of OMT treatment protocols on the dysarthria presented in CP patients. Finally, an instrumental dynamic study such as VFSS before and after OMT and respiratory therapy regimens to note more objectively the effects of feeding and swallowing parameters could be of great relevance.

## 5. Conclusions

Children who received CP during the last OMT, in some of its modalities, showed better FSS, regardless of their level of gross motor functionality, as well as better indicators of nutritional status and less frequency of respiratory diseases, which results in important benefits for the health of this population group.

This is the reason why we propose that OMT be included in the rehabilitation intervention schemes for children with CP since the benefits indicated in this study highlight the importance of FSS in the health and quality of life of children with CP. It would also be important to evaluate the different OMT techniques and establish the clinical benefits associated with each of them and standardize a management scheme for children with CP.

## Figures and Tables

**Table 1:** Feeding oral motor scale.

Skill	Dimensions
Posture	Head-neck control and alignment
	Head mobility
	Scapular mobility
	Trunk stability

Jaw mobility	Lateral
	Opening-closing
	Movement coordination

Tongue activity	Lateral
	Retraction
	Up and out movement
	Rest position

Lips	Lip seal
	Lip protrusion
	Rest position

Feeding behavior	Liquid suction
	Chewing
	Swallowing

Abnormal reflexes	Phasic biting
	Gagging

Breathing control	Breath-swallow coordination

General oral motor skills	—

**Table 2:** Description of PC types and motor impairment in the groups with and without OMT.

Groups	Clinical type: *N*	Topographic type: *N*	Gross motor level: *N*
With OMT, *N*:15	Ataxic: 2	Diplegia: 4	High function: 5
	Spastic: 9		
	Hypotonic: 2	Hemiplegia: 1	Low function: 10
	Mixed: 2	Tetraplegia: 10	

Without OMT, *N*: 15	Spastic: 11	Diplegia: 1	High function: 6
	Hypotonic: 2	Hemiplegia: 2	Low function: 9
	Mixed: 2	Tetraplegia: 12	

This chart shows that both groups had similar PC types and motor conditions (chi squared, *p* = 0.7).

**Table 3:** Feeding and swallowing skills in Yucatecan children with CP.

Skills	With OMT	Without OMT	*p*
Posture	6.00 ± 0.640	7.40 ± 0.804	0.184
Jaw mobility	4.27 ± 0.358	5.47 ± 0.350	0.023^∗^
Tongue activity	5.73 ± 0.228	6.87 ± 0.496	0.047^∗^
Lips	4.13 ± 0.307	5.00 ± 0.352	0.074
Feeding behavior	3.60 ± 0.190	4.33 ± 0.410	0.116
Abnormal reflexes	0.00	0.27 ± 0.118	0.032^∗^
Breathing control	1.00	1.27 ± 0.118	0.032^∗^
General oral motor skills	24.73 ± 1.240	30.60 ± 2.190	0.027^∗^

Values represent mean ± standard deviation. Student's *t*-test for independent samples. ^∗^Significant difference.

**Table 4:** Relation between OMT and gross motor.

Gross motor level (GMFCS)	With OMT	Without OMT	*p*
High function levels (II and III)	20.80 ± 1.304 (*n* = 5)	22.33 ± 1.033 (*n* = 6)	0.028^∗^
Low function levels (IV and V)	26.70 ± 4.715 (*n* = 10)	36.11 ± 6.314 (*n* = 9)	0.003^∗^

Values represent mean ± standard deviation, while the correspondent *n* is in parenthesis. Student's *t*-test for independent samples.

## Data Availability

The datasets were obtained by the authors.
